# Continuous volitional control of a bionic leg supports diverse walking patterns in both agonist–antagonist muscle interface and bone-anchored prosthesis users

**DOI:** 10.1093/pnasnexus/pgaf413

**Published:** 2026-01-05

**Authors:** Federica Damonte, Lucas Avanci Gaudio, Jose Gonzalez-Vargas, Guillaume Durandau, Jennifer Ernst, Johan S Rietman, Ruud Leijendekkers, Herman van der Kooij, Massimo Sartori

**Affiliations:** Department of Biomechanical Engineering, University of Twente, Enschede 7522 LW, The Netherlands; Global Research, Ottobock SE & Co. KGaA, Duderstadt D-37115, Germany; Department of Biomechanical Engineering, University of Twente, Enschede 7522 LW, The Netherlands; Global Research, Ottobock SE & Co. KGaA, Duderstadt D-37115, Germany; Global Research, Ottobock SE & Co. KGaA, Duderstadt D-37115, Germany; Department of Mechanical Engineering, McGill University, Montreal, QC, Canada H3A 0C3; Department of Trauma Surgery, Hannover Medical School, Hannover OE-6320, Germany; Department of Biomechanical Engineering, University of Twente, Enschede 7522 LW, The Netherlands; Roessingh Research and Development, Revalidatiecentrum Roessingh, Enschede 7522 AH, The Netherlands; Department of Rehabilitation, Radboudumc, Nijmegen 6525 GA, The Netherlands; Department of Biomechanical Engineering, University of Twente, Enschede 7522 LW, The Netherlands; Department of Biomechanical Engineering, University of Twente, Enschede 7522 LW, The Netherlands

**Keywords:** bionic limbs, EMG-driven musculoskeletal model, leg amputation, neural interface, volitional control

## Abstract

Myoelectric control paradigms have the potential to enable continuous volitional control of bionic limbs in various movement conditions. Although individuals with below knee amputations and an agonist–antagonist muscle interface (AMI) were proven to display a greater degree of continuous volitional control in bionic ankle-foot systems with respect to conventional socket-suspended prosthetic users, it remains unclear how myoelectric interfaces could translate to non-AMI prosthetic users with bone-anchored prostheses (BAP). This preliminary study proposes a human–machine interface (HMI) based on a neuromechanical model to enable volitional, continuous myoelectric control of a bionic leg in AMI and BAP users, walking across various speeds and ground inclinations. Differently from state of the art solutions, the proposed HMI is based on a digital twin of the intact leg, synthesizing the user’s phantom limb musculoskeletal function as controlled by muscle activations measured from the residuum. When embedded in a real-time framework, it enabled the participants to achieve volitional modulation of prosthesis peak plantar-dorsiflexion torques timing and amplitude during overground walking at three speeds (between 1.6 and 3.96 km/h), with case studies provided during calf-raises (30, 45, and 60 bpm) and ramp ascent walking (3 and 5% incline). Before prosthesis control tests, the participants underwent a 2-day gait training session. Results showed that all three subjects learned how to alter initial muscle activation patterns so that an average of 87% of peak activation timing fell within target ranges. The proposed neuromechanical modeling technology opens new avenues toward generalizable HMIs for the volitional control of active prostheses beyond set conditions and amputation types.

Significance Statement Lower-limb amputees often lack intuitive volitional control over powered prosthetic legs, especially across different walking speeds and ground elevations. We introduce a human–machine interface that enables continuous, volitional control of a bionic ankle-foot prosthesis by translating residual muscle activity into biomechanically meaningful joint torques using a personalized digital twin of the user’s intact leg. We demonstrate this approach in agonist–antagonist muscle interface and bone-anchored prosthesis users during walking at multiple speeds, inclines, and during weight-bearing tasks. After brief training, participants learned to modulate muscle activation timing and amplitude to control prosthetic joint torques with no need for state machines or rule-based controllers. These results show that our human-machine interface, based on an electromyography-driven musculoskeletal model, can generalize myoelectric control across movements and amputation types, representing a step toward more natural, adaptive, and user-driven bionic limbs.

## Introduction

Human movement underlies interaction and coordination across nervous, muscular, and skeletal systems. Thousands of microscale neural structures, such as spinal motor neurons are recruited to form the final neural code instructing skeletal muscles how to contract for the execution of a movement (1, 2). Recruited fibers collectively generate mechanical forces, which are subsequently transferred via series-elastic tendons to the skeletal system, thereby accelerating biological joints (2). These neuromuscular mechanisms are key for producing the broad range of biological joint torques that accelerate anatomical limbs and enable functional movements such as locomotion across different speeds (3).

A transtibial amputation disrupts the neuromuscular pathways underlying the closed-loop control of the ankle muscles (4). However, motorized prostheses (or bionic limbs), are emerging as a viable solution to replace the biomechanical function of the missing limb (5). A key challenge lies in designing human–machine interfaces (HMIs) that allow individuals who underwent diverse types of amputation procedures to gain volitional control of their bionic limbs, as a natural extension of their own body in a task-agnostic manner (5, 6).

Despite advances in electromyography (EMG)-based control and surgical techniques like targeted muscle reinnervation (TMR) and agonist–antagonist muscle interfaces (AMIs), bionic legs remain limited in terms of clinical and commercial impact (7–10). Available HMIs often rely on rule-based algorithms, such as finite state machines, which impose predefined impedance properties based on detected motor tasks and gait phases (5). However, these predefined behaviors often fail to match actual movement demands and do not allow users to continuously modulate torque in response to changes in speed, terrain, or perturbations (9, 11).

Human intent recognition for bionic legs has primarily relied on surface EMGs in the form of discrete classifiers, offering a viable way to interface with the neuromuscular system (5, 12). While EMG classifiers can detect discrete locomotion modes, they limit the user’s ability to volitionally and continuously control prosthetic limb dynamics in real time (13, 14). A myoelectric solution was proposed for the continuous and proportional control of bionic leg plantar-dorsiflexion during walking, stair climbing (15) or perturbed standing (13, 16), with bionic leg dorsiflexion being automatically engaged when the participant’s plantarflexor muscles relaxed (17, 18). Recent work utilized EMG signals from an AMI established between the residual limb’s tibialis anterior and gastrocnemius muscles, to estimate a target joint position and impedance, while modulating maximum joint torques based on joint angle and velocity (19). More recent studies have explored continuous myoelectric control outside laboratory environments, demonstrating that transtibial amputees with socket-suspended bionic legs could volitionally activate their residual limb muscles to control bionic ankle in real-world settings (20).

However, while bionic leg users who underwent the AMI surgery have so far displayed increased degree of volitional myoelectric control across various locomotion tasks (19) with respect to non-AMI individuals with traditional socket-suspended prostheses (18, 20, 21), it remains unclear how myoelectric control paradigms for bionic legs would translate to non-AMI bionic leg users with bone-anchored prostheses (BAP) (22, 23). In BAP individuals, the absence of the suspended socket would likely alleviate the need for residuum muscle contractions aimed at socket stabilization, which would otherwise yield to sub-optimal muscle activation for myoelectric controllers (24). Moreover, BAP users have displayed improved walking performance, with faster walking speeds, with respect to conventional non-BAP, socket-suspended prosthetic users (24). However, differently from AMI-amputees, in BAP-amputees, sensory feedback from the muscles is not restored.

In this article, we propose a myoelectric HMI based on an EMG-driven neuromechanical model, hereafter referred to as the neuromechanical model-based control (NMBC) paradigm (25). Specifically, we investigate how NMBC could be used to enable continuous and volitional myoelectric control of bionic legs across locomotion speeds and ground-inclines in three individuals with transtibial amputation with AMI and BAP procedures, respectively i.e. in two individuals who underwent the AMI surgical procedure that used a socket-suspended bionic leg (8, 26–28) and in one individual who used a BAP bionic leg with a press-fit osseointegration implant (BADAL X OTI, OTN Implants BV, NL) (29).

The proposed NMBC relies on real-time EMG-driven musculoskeletal models of the phantom limb, which are used to convert residuum muscle EMGs into bionic limb torque commands (30). This allows for the continuous EMG decoding of biomechanically plausible joint torques, which enable the bionic leg to closely mimic the biomechanical function of intact limbs during dynamic weight-bearing locomotion (i.e. speeds between 1.6 and 3.96 km/h and 0, 3, and 5% inclines). Differently, from state of the art work, the proposed NMBC incorporates a musculoskeletal model of the phantom limb, which was created as the digital copy of the participant’s intact contralateral limb (30). That is, NMBC’s person- and leg-specific EMG-driven musculoskeletal model captured multiple biomechanical properties of the participant‘s intact leg, including limb anthropometry, and musculoskeletal geometry, as well as the force-generating capacity of the major muscle-tendon units (MTUs) spanning the ankle joint.

Previous studies have employed various approaches to drive and calibrate musculoskeletal models. Several studies relied on normalized EMGs alone, which in turn were used to modulate the setpoint of motor control variables (18, 20). Other studies have explicitly utilized musculoskeletal models to generate prosthesis commands (6, 21), leveraging simulated muscles and skeletal geometries to determine joint-level torques. In Ref. (21), the musculoskeletal model was calibrated for each individual using a simulated reflex model that incorporates the participant’s body characteristics. In contrast, Ref. (6) derived muscle parameters through data-driven optimization based on healthy individuals. However, no study has yet developed musculoskeletal models that explicitly match the anatomical, geometric, and force-generating properties of the prosthesis user’s intact limb in AMI and BAP individuals.

Our results showed that, following a 2-day visual feedback-based training, the proposed approach enabled fully volitional control of a motorized ankle bionic leg, simultaneously in plantar and dorsiflexion, across weight-bearing walking at three speeds (Results section) and two ground-incline levels (Supplementary material). Results showed that bionic limb volitional control could be achieved throughout the entire movement cycle with no need to use a priori defined finite state machines.

## Materials and methods

### Experimental protocol

We enrolled three male participants (subject 1-AMI: age 65, weight 103 kg, height 196 cm; subject 2-AMI: age 31, height 189 cm, weight 85 kg; subject 3-BAP: age 47, height 185 cm, weight 85 kg), with below-knee amputation, who volunteered for this experiment and gave their informed written consent. Subject 1-AMI underwent an AMI surgical procedure (8) combined with TSR (10) of the medial skin of the residual limb by the sural nerve to restore sensation of the foot sole; subject 2-AMI underwent an AMI surgical procedure, and subject 3-BAP underwent an osseointegration procedure. The project was approved by the Central Committee on Research Involving Human Subjects (Ministry of Health, Welfare and Sport of Oost-Nederland - WMO), dossier number NL81380.091.22.

**Fig. 1.**
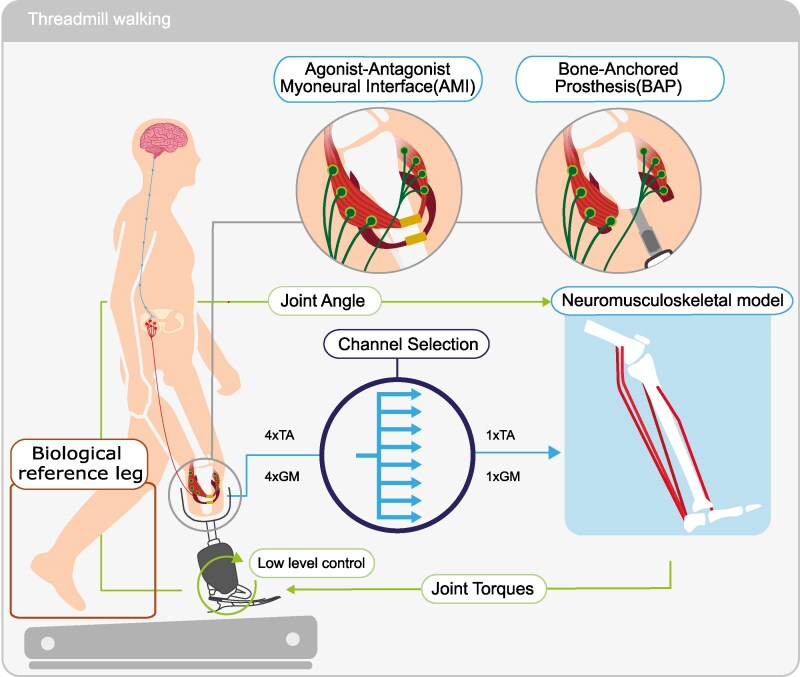
Overview of the EMG-driven neuromusculoskeletal model integration with the bionic leg. Two of the eight bipolar channels placed on top of both gastrocnemius medialis (GM) and tibialis anterior (TA) muscles were selected to drive the neuromusculoskeletal model (more details in the Materials and methods section). Joint angles from the bionic leg were fed back to update the kinematic-dependent states of the neuromusculoskeletal model (e.g. muscle-tendon unit length and moment arms), which outputs reference control torques to the bionic leg low-level controller (more details in Materials and methods—Software and hardware control pipeline section). Model parameters were obtained via a data-driven calibration, which aimed at identifying intact leg’s anthropometry, musculoskeletal geometry, and force-generating parameters (more details in Materials and methods—NMBC calibration section). Three participants with either agonist–antagonist myoneural interfaces (AMI) or bone-anchored prosthesis (BAP) amputations performed treadmill walking at different speeds and inclines.

Participants completed a 3-day session at the University of Twente (Enschede, The Netherlands—Fig. 1). Day 1 covered electrode placement, device alignment, and EMG-biofeedback training; day 2 included calibration and initial control of the active prosthesis, and day 3 comprised of treadmill and calf-raise trials using personalized neuromechanical controllers. Full protocol details and per-subject conditions are provided in the Supplementary material, Section 11.1.

### Software and hardware control pipeline

An active ankle bionic leg capable of plantar-dorsiflexion (EMPOWER, Ottobock SE & Co. KGaA, Duderstadt, Germany), operated in current control mode and received motor current commands. The maximum allowed current was equivalent to a torque of 50 Nm (i.e. for subject 1-AMI and 2-AMI) and 70 Nm (i.e. for subject 3-BAP) in isometric conditions at the neutral foot position (refer to Section 11.5.1). An embedded interface hardware (NUCLEO-F767ZI, STMicroelectronics, Geneva, Switzerland, and netSHIELD NSHIELD 52-RE, Hilscher Gesellschaft für System automation mbH, Hattersheim am Main, Germany) converted incoming torque commands into current setpoints by dividing them by the motor constant ($k_{t}$) adjusted by the motor-to-joint gear ratio. The embedded interface received commands through an Ethernet for Control Automation Technology (EtherCAT) communication protocol. The embedded hardware was also responsible for making available internal data from the bionic leg, such as joint angles. A workstation (Windows 10 Pro) ran a communication and processing master (TwinCAT, Beckhoff Automation GmbH & Co. KG, Verl, Germany) as well as a neuromusculoskeletal toolbox (developed using CEINMS-RT (31)), responsible for simulating the participant-specific EMG-driven musculoskeletal model, which in turn generated prosthetic torque setpoints. Data collected during the experiment, except for marker data, as described in the Data collection section, were also aggregated into the TwinCAT master.

Additional software running in the TwinCAT master was also capable of offsetting setpoint torques to the bionic leg. Such offsets moved torque biases as outputs of the muscle model back to zero.

### Neuromechanical modeling

We used the open-source CEINMS-RT platform previously developed by the authors to create the NMBC mid-level bionic leg controller based (1, 25, 31, 32). This consisted of a personalized (see Model calibration section) EMG-driven-musculoskeletal model capable of predicting joint torques in real-time (31). Supplementary material includes a detailed schematic of the control pipeline designed for the bionic leg (see Fig. S1). Real-time muscle EMG signals were filtered (see Online data processing pipeline section) and normalized to the maximal voluntary contraction (MVC) value. MVC was obtained while seated and while standing after each electrode replacement. The recorded EMGs from the gastrocnemius medialis (GM) drove 3 MTUs in the model (stage B, Fig. S1): GM, gastrocnemius lateralis (GL), and soleus (SO), which were modeled as synergistic (33). Muscle activations of the tibialis anterior (TA) drove the respective MTU counterpart in the model. The internal encoder of the bionic leg provided the ankle joint angles, which were used as input to cubic B-splines, derived from a participant-specific OpenSim geometry model (stage C, Fig. S1) (34). Joint angle-driven B-splines were used to compute MTU’s muscle-tendon length (LMT) as well as moment arms (MA) (35). Since the knee angle measurements were not available during the real-time experiment, the knee joint was assumed to be fully extended. This specific angle was chosen because the gastrocnemii force-contribution is more pronounced at extended knee angles.

Finally, in stage D—Fig. S1, the muscle activation signals and MTU lengths were used to compute the muscle-tendon forces using a Hill-type model (36, 37). The joint torque at the ankle was the result of the projection of MTU forces via MA (stage E—Fig. S1).

### NMBC calibration

The open-source software OpenSim (38) was utilized to adjust a generic musculoskeletal geometry model (32) to match each participant’s anthropometric data. We employed a generic full-body model of the musculoskeletal geometry to represent the participant’s anthropometry (gait2392 model (38)). Joint positions, center of mass, force application points, MTUs, and ligament attachment points were linearly scaled according to the distances between the experimental and virtual markers (32). Although the full-body model was scaled, the calibration procedure involved 4 MTUs and 1 degree of freedom (DOF) around the ankle joint.

The NMBC optimization algorithm (32) requires as input the EMG signals from the residual limb, along with joint angles derived via inverse kinematics as well as joint torques derived via inverse dynamics, referred to as the “experimental reference joint torques” from the intact side. To ensure temporal alignment between limbs, the EMG signals were shifted to match the movement of the intact leg. In this step, an extended set of musculoskeletal parameters, including the MTU excitation-to-activation shape factor, MTU strength coefficients, tendon slack length, and optimal fiber length, was calibrated to match the ankle plantar–dorsiflexion torque profile of the intact leg over multiple complete gait cycles at the subject’s preferred walking speed. Experimental findings showed that the biological dorsi-flexion torques derived via inverse dynamics, when used to drive the prosthesis, were not sufficient to dorsiflex the prosthesis at toe off. We hypothesized this was due to internal friction within the prosthesis drivetrain. Therefore, reference ankle dorsiflexion torques used in calibration were artificially increased during early swing to account for the additional torque required to overcome this friction. The first positive half-cycle of a sinusoidal curve was manually added to the experimental joint torque (at 65–85% gait phase) with a peak amplitude of 15 Nm. The calibration was performed offline, and no additional state machine was used to inform the controller during real-time myoelectric control tests.

The calibration of NMBC’s inner MTU model force-generating parameters was based on two optimization steps, which enabled the identification of parameters that vary nonlinearly with individuals’ anthropometries (31). In the first optimization step, we derived initial values for tendon slack lengths and optimal fiber lengths within each modeled MTU. This was done as previously described to preserve normalized values between generic and linearly scaled musculoskeletal geometries (39). In the second optimization step, an extended parameter set was calibrated including the MTU excitation-to-activation shape factor, MTU strength coefficients, tendon slack length, and, optimal fiber length. A simulated annealing algorithm was used to vary the above-listed parameters, minimizing the sum of the mean square differences between the predicted and experimental joint torques calculated over all calibration trials (32).

### Data collection and processing

Details on data collection and processing are presented in the Supplementary material, Sections 11.4, 11.5.1, and 11.5.2.

## Results

### Bionic leg volitional control across ground-level treadmill walking speeds

**Fig. 2.**
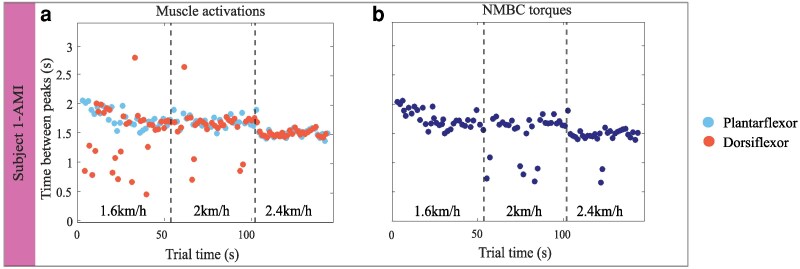
Time in seconds between peaks of muscle activations for gastrocnemius medialis (GM) (blue) and tibialis anterior (TA) (red) (a) and the neuromechanical model-based control (NMBC) torques (b), during the walking trial on a treadmill on the second day of experimental sessions of subject 1-AMI. Peaks were obtained by filtering the signal and detecting local maxima. Only peaks above a threshold value were considered and therefore located at a specific time (s) during the trial.

**Fig. 3.**
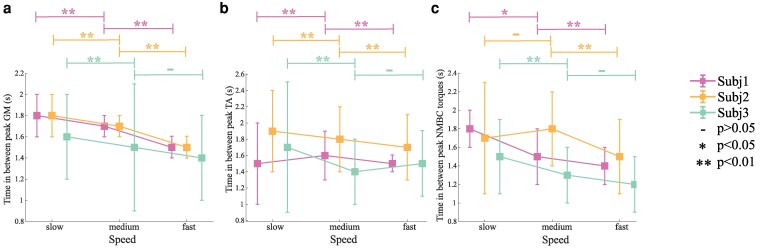
Average and SD of the time, in seconds, between peaks of gastrocnemius medialis (GM) activation (a), tibialis anterior (TA) activation (b), and the neuromechanical model-based control (NMBC) plantarflexion torque (c) for each different speed and subject.

Results showed the participants could always modulate bionic leg peak torque timing across all walking speeds (Figs. 2 and 3). In this context, we analyzed timing in between consecutive muscle activation peaks (a) and, plantar-dorsiflexion torque peaks (b) throughout the entire test duration (Figs. 2 and S2).

Overall, for the residual GM muscle, the time between muscle activation peaks decreased proportionally to the increasing speeds for the three subjects (see Tables S1–S3). On the other hand, considering the residual TA muscle, the time between muscle activations does not show a pattern with gait speed. Only subject 2-AMI could modulate the muscle activations proportionally to speed resulting in a decrease of time between activation peaks (see Tables S1–S3). Figure 3a displays observed modulations in muscle activation patterns. During the 1.6 km/h speed condition, subject 1-AMI progressively adjusted step duration to gradually reduce step time, thereby leading to muscle activation timings at the end of the walking trial more similar to those observed during self-selected speed (2 km/h, Fig. 2a and b) i.e. step duration transitioned from 1.93 s at the beginning of the trial, to 1.56 s at the end. In contrast, subjects 2-AMI and 3-BAP showed a more distinctive step duration across speeds: step duration transitioned from 2.1 s to 1.7 s and from 1.52 s to 1.31 s from low to fast speed, respectively. Figure S2 shows the observed variations in muscle activation patterns and joint torques for these two subjects.

During the trials, all subjects activated each muscle multiple times throughout the gait cycle, leading to secondary activation peaks and resulting in outliers with short intervals (Figs. 2a–S2), consequently pushing the average time between peaks to a lower value than expected (Figs. 2a–S2). Peak activations for both GM and TA manifested within phases of the gait cycle comparable with those reported in the literature from intact-limbed individuals: 30–50% and 0–10% and 80–90%, respectively (2). Considering subject 1-AMI, peak GM activations were located at an average of 42.5% within the gait cycle, while TA activations were located at 81.3%. Similarly, subject 2-AMI showed peak GM activations at 39.5% and peak TA activations at 60% of the gait cycle. In contrast, peak GM activations of subject 3-BAP were located at 59.9% within the gait cycle and, peak TA activations were located at 62.6%. The peak NMBC torques manifested at *(mean ± SD)*  47.6±11.8, 46.0±13.1, and 49.1±11.0%; 40±15, 41.0±14.9, and 38±20.0%; 65.5±5.2, 61.8±17.3, and 66.5±17.6% of the gait cycle for the increasing walking speeds, for subject 1-AMI, subject 2-AMI and, subject 3-BAP respectively. Decoded torques were limited to 50 or 70 Nm as a safety measure for bionic leg control (more details in Methods-Online data processing pipeline section), which in turn resulted in a flat section in the torque profiles of Fig. S3.

### Gait training via muscle visual feedback

**Fig. 4.**
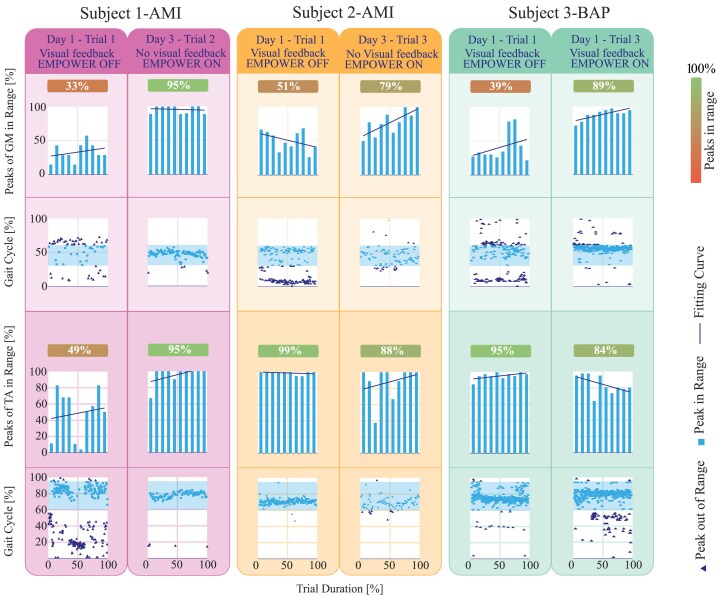
Histograms and scatter plots for locomotion trials used for training. Each scatter–histogram pair corresponds to a single locomotion trial. The top pair shows data from the gastrocnemius medialis (GM), and the bottom pair shows data from the tibialis anterior (TA). Visual feedback was provided during the first two experimental days, during which the bionic leg was powered OFF for all participants. On the final day, visual feedback was removed and the bionic leg was powered ON. Scatter plots show the percentage of the gait cycle at which peak EMG activations of GM and TA occur for each gait cycle within a trial. The *x*-axis represents the normalized trial duration. Shaded regions indicate the expected range of peak activation in healthy gait (30–60% of the gait cycle for GM and 60–95% for TA). Light-colored squares denote peak activations within the expected range, whereas dark-colored triangles denote peaks outside this range. Histograms show the percentage of peak activations within the expected range for each decile of the trial duration. A first-degree polynomial fit is overlaid (blue line).

Figure 4 describes the evolution of peak activations for each subject, from the first trial to a consecutive one, which shows the best performance achieved during the three days of the experiment. The plots in the first two rows of Fig. 4 show the percentage of activation peaks for the GM muscle. Regarding subject 1-AMI, the peaks within the target range increased significantly from 33% (day 1, trial 1-EMPOWER OFF) to 79% (day 2, trial 1-EMPOWER OFF), to then decrease again to 47% (day 2, trial 2-EMPOWER OFF). On day 3, training was performed with the bionic leg powered ON. The percentage of peaks within the target range increased rapidly, starting at 69% (day 3, trial 1) to then reaching 95% (day 3, trial 2). Similarly, for subject 2-AMI, the peaks within the target range increased from 51% (day 1, trial 1-EMPOWER OFF) to 79% (day 2, trial 1-EMPOWER ON), to then decrease again to 52% (day 3, trial 1). On day 3 the percentage of peaks within the target range increased from 59% to then reaching 79% (day 3, trial 3). Finally, considering subject 3-BAP, the peaks within the target range increased from 39% (day 1, trial 1-EMPOWER OFF) to 94% (day 1, trial 5-EMPOWER ON). On day 3, the percentage of peaks within the target range decreased compared to day 1, reaching an average of 21%. The mean value of GM peak activation for the three subjects stabilized at 48% of the gait phase, which was within the acceptable range for a symmetric walking pattern (18). The last two rows of Fig. 4 show the percentage of peaks within the target range for the TA muscle. Results for subject 1-AMI showed that peaks within the target range increased significantly from 49% (day 1, trial 1-EMPOWER OFF) to 96% (day 2, trial 1-EMPOWER OFF), to then decrease again to 81% (day 2, trial 2) for TA. On day 3, results showed that the percentage of peaks within the target range increased, starting at 90% (day 3, trial 1) to then reaching 95% (day 3, trial 2). In contrast, for subject 2-AMI, the peaks within the target range decreased from 99% (day 1, trial 1-EMPOWER OFF) to 45% (day 2, trial 1-EMPOWER ON), to then increase again to 92% (day 3, trial 1-EMPOWER ON). On day 3 the percentage of peaks within the target range decreased from 92% to then reaching 88% (day 3, trial 3). Finally, considering subject 3-BAP, the peaks within the target range decreased from 95% (day 1, trial 1-EMPOWER OFF) to 7% (day 1, trial 5-EMPOWER ON). On day 3, the percentage of peaks within the target range decreased from 86% (day 3, trial 1) to 53% (day 3, trial 3). The mean value of TA peak activation for the three subjects stabilized at 73.1% of the gait phase, which was within an acceptable range to guarantee enough dorsiflexion that generates sufficient toe clearance (18). Furthermore, for both muscles, within each of the trials, it was observable that the number of peaks in the range tended to rise as the participant got used to that walking condition. The shape of both GM and TA activation patterns changed throughout the trials, where the GM and TA peak activations stabilized within expected targets (2). Correlation values *R* associated with healthy activation patterns from literature (40) indicated a change in activation shape across days (see Table S4). Subjects 1-AMI and 2-AMI demonstrated an increase in GM correlation and a decrease in TA correlation, while subject 3-BAP exhibited the opposite pattern.

### Bionic leg volitional control during treadmill walking at different ground inclinations

Results showed that subject 2-AMI and subject 3-BAP could modulate active bionic leg peak torque amplitude across all inclinations (Fig. S4). In this context, we analyzed the amplitude of muscle activation peaks and bionic leg plantar torque peaks throughout the entire test duration (Tables S5 and S6).

For the residual GM muscle, the amplitudes of muscle activation peaks were (mean±SD) 0.32±0.17, 0.38±0.12, and 0.38±0.12, 0.53±0.29, 0.23±0.23, and 0.18±0.28 for subject 2-AMI and subject 3-BAP, respectively, at the ground, 3%, 5% inclinations (Fig. S5). For the residual TA muscle, the amplitude of muscle activations was 0.32±0.17, 0.38±0.12, and 0.63±0.19, and 0.18±0.12, 0.25±0.22, and 0.09±0.13 for ground, 3%, and 5% inclinations, for subject 2-AMI and subject 3-BAP, respectively (Fig. S5).

Figure S4 displays observed modulations in computed NMBC and active bionic leg torques.

The amplitude of the active bionic leg peak torques decoded by the proposed musculoskeletal model was modulated as a function of walking inclinations. Peak plantarflexion torque values were *(mean ± SD)*  −29.5±17.4, −37.7±16, and −40.7±17.1 Nm and, −35.2±23.7, −14.2±8.2, and −29.1±26.3 Nm, for ground, 3% and 5% inclinations, for subject 2-AMI and, subject 3-BAP, respectively. These manifested at *(mean ± SD)*  41.05±14.9, 40.4±13.9, and 35.5±12.08%; 61.8±17.32, 59.8±9.95, and 58.5±8.02% of the gait cycle for the increasing inclination, for subject 2-AMI and subject 3-BAP, respectively. Decoded torques were limited to 50 or 70 Nm as a safety measure for bionic leg control (more details in Methods-Online data processing pipeline section).

### Bionic leg volitional control during calf raise

Figure S6a shows timing distribution between consecutive peaks of muscle activations, bionic leg joint peak plantarflexion torque, and its respective peak velocity during the calf raise test. The three tested cadences, 30, 45, and 60 beats per minute (bpm), underlay time between beats of 2, 1.33, and 1 s, respectively. Subject 1-AMI was unable to follow the 60 bpm pace.

Timing in between muscle activation peaks decreased with the increasing frequency. Muscle activations and bionic leg plantarflexion torque peak timing never deviated from expected timings by more than 0.1 s. That is, average and SD of time in between muscle activation peaks were 2±0.1, 1.4±0.1 for the 30 and 45 bpm, respectively; average and SD of time in between plantarflexion torque peaks were 2±0.5, 1.4±0.1 for the 30 and 45 bpm, respectively (Table S7). Peak plantarflexion joint velocity displayed average timings of 2.7±3.5s and 1.6±1.2s for the 45 bpm and 30 bpm cadences, respectively. Timing deviations in joint velocity peaks were contaminated by one outlier lying at 20 s for the 30 bpm cadence, and at 8.5 s for the 45 bpm trial (Fig. S6a bottom histogram).

Table S7 shows a comprehensive overview of timing values.

Figure S6 illustrates GM and TA muscle activations (b and c), respectively, joint plantarflexion torques (d), and ankle plantarflexion angles (e) during bionic leg trial during calf-raise.

The amplitude of the peak activation of TA and GM translated into comparable modulations in torque (d) and joint angles (e).

## Discussion

We presented an HMI paradigm (Fig. 1), where the information extracted from an individual’s neuromusculoskeletal system (i.e. from measured muscle activations to decoded phantom ankle joint mechanics, Figs. 2 and S3) was used to enable volitional control of a motorized bionic leg (Videos S1 to S8). We tested this paradigm in two individuals with transtibial amputation who underwent the AMI surgical procedures and in one additional BAP individual with transtibial amputation. Differently from previous work, this study proposed the development of the NMBC myoelectric model-based control framework that enabled continuous and volitional control of a bionic leg. These results provide preliminary indications that the proposed NMBC framework may enable myoelectric control across a range of movements (i.e. three walking speeds, three ground inclinations, and two calf rise paces) as well as across amputation types not previously compared within a single study (i.e. AMI and BAP procedures). However, given the small sample size (n=1 for BAP, n=2 for AMI), these findings cannot be generalized. Importantly, the proposed NMBC involved no use of finite-state machines, thereby enabling full continuous and volitional control (Fig. S3). Unlike previous literature, the proposed NMBC incorporated a digital twin of the participant’s phantom leg that was numerically built to match the anthropometry, musculoskeletal geometry, and force-generating capacity of the participant’s intact leg (9, 30). This provides a systematic way of establishing EMG-driven musculoskeletal phantom limb models, differently from previously proposed approaches, where the musculoskeletal model’s internal parameters were not numerically identified directly from the amputee’s residual musculoskeletal system (6, 18, 20, 21). Additionally, despite day-to-day variations in electrode placement, our framework allowed us to utilize the same NMBC subject-specific calibrated model without further model adjustments, which could be advantageous for the use of this type of control in clinical settings.

In able-bodied individuals, biological joint torque peaks are volitionally modulated in both amplitude and timing across different walking speeds (2). At high walking speeds, gait cycle duration can be <1.5 s (41). This represents a short time window for precise joint peak torque modulation, which poses challenges for bionic leg myoelectric control. During our proposed walking tests, the ability to control bionic leg peak torque timing volitionally was critical for cyclic and stable gait across different speeds (Figs. 3, S3, and S2). In our study, results showed that both AMI and BAP participants dynamically modulated the bionic leg torque to movement conditions imposed by the treadmill belt motion for three different speeds (see Fig. 3, Video S1). For different walking speeds, participants consistently modulated the timing of GM activations (Fig. 3a), with activation periods becoming shorter as speed increased. A difficulty in activating the TA consistently skewed the results of subjects 1-AMI and 3-BAP, preventing TA activations from displaying the expected descending pattern observed in GM. Activations of TA followed the same pattern as GM for subject 2-AMI. In this context, participants 1-AMI and 3-BAP could volitionally command torque profiles to the bionic leg with decreased timing between plantarflexion torque peaks as walking speed increased (Tables S1 and S3), respectively. Subject 2-AMI demonstrated an increase in the torque peak period between slow and medium speed and a reduction between the medium and fast speeds. Torque commands generated by the implemented NMBC arose from the opposing actions of antagonistic muscle pairs, their activation levels, and relative isometric forces. The delayed activations of the TA influenced torque peaks for subject 2-AMI at a walking speed of 2.34 km/h, causing a delayed dorsiflexion, which resulted in longer torque peak periods and disrupted the expected trend. Nevertheless, our proposed human–machine interface enabled the participant to accurately control the timing of the bionic leg peak torques in a brief time window corresponding to the gait cycle time. Future studies should tailor the duration of the training to reach a minimum percentage of activations within range for both GM and TA.

Commanded peak torques exhibited no consistent pattern across subjects. Higher torques were observed when deviating in either direction (both higher and lower speeds) from the self-selected speed for subject 1-AMI, while subject 2-AMI displayed the highest torque at the self-selected speed. In contrast, torque decreased with increasing speed for subject 3-BAP. In intact-limb indviduals (42), torque peaks increase with speed. However, the onset of joint torque within the gait cycle fell within the expected range for all subjects and speeds.

AMI subjects improved the control of GM even when visual feedback of muscle activations was removed, as shown in Fig. 4. In contrast, subject 3-BAP showed activation timings matching those expected in over 95% of gait cycles during trials with visual feedback (Fig. 4) but showed a significant decline in expected GM activations when it was absent. This suggests that maintaining focus on the movement could be essential for his performance. Future studies should explore whether the inclusion of extended training sessions for subjects with BAP is beneficial and further understand the discrepancy between the training and walking test performances. Looking at the NMBC torques generated (Fig. S3), AMI subjects showed fine control of the torque during stance, more gradual, while subject 3-BAP showed an on-off ballistic behavior, likely resulting from the reduced proprioceptive feedback of this participant. Indeed, literature showed that people with conventional amputations (no AMI or TSR procedures) often experience difficulty in finely modulating residual muscle activation, which results in abrupt activation–relaxation patterns (43).

Subjects 1-AMI and 2-AMI exhibited GM activation patterns comparable to those of intact-limbed individuals (Table S4). In contrast, subject 3-BAP faced challenges in activating GM, with post-training *r*-values being lower than *p*-training values (Table S4). The shape of the activation patterns of TA did not closely align with those observed in intact-limb individuals for any of the subjects.

Walking on uphill inclined ramps is an important activity of daily living that can be challenging for individuals with amputations. Indeed, walking uphill requires an increased range of motion of the ankle joint compared to level-ground walking, increasing the activity of GM and decreasing TA (44). Looking at the volitional control during walking on an inclined treadmill, both subject 2-AMI and subject 3-BAP generated different amplitudes of muscle activations for each inclination. Subject 2-AMI managed to increase the activation amplitude of GM proportionally to the inclinations as shown in healthy subjects (2, 44), the activity of TA also showed an increased activity which is instead not natural. On the other hand, for subject 3-BAP only TA showed a decreased activity from 3% to 5% elevation (Fig. S5) as shown in healthy subjects (2, 44). The performance of subject 2-AMI degraded compared to the ground-level walking, the torque shape resulted to be more irregular may be due to the increased socket pressures at the calf muscles (45). Moreover, co-contractions increased perhaps to create suspension while the prosthetic socket undergoes a higher distraction force from the residual limb during the initiation of swing (46). On the other hand, subject 3-BAP showed a ballistic type of control as in the overground walking, reducing GM-TA co-contractions (see Fig. S4).

Calf raise tests showed that subject 1-AMI was able to volitionally modulate the timing of the bionic leg plantar-dorsiflexion torque when the residual limb was loaded with the participant’s weight, therefore exercising a functional role of weight-bearing and vertical propulsion (see Fig. S6, Videos S2 and S3).

Gait training was demonstrated to be an important step in guaranteeing adequate learning of bionic leg torque control for people with conventional amputation (18). However, no evidence in previous literature showed how, and if, a training session should be carried out in the case of participants with AMI, which motivated the inclusion of a training session in our experiments (Fig. 4, Results-Gait training via muscle visual feedback section, and Video S4). Our results indicate that the training sessions with visual feedback are likely to help AMI participants improve their performances of GM activation, supporting conclusions from prior studies: individuals with transtibial amputation who underwent AMI could be trained to generate muscle activations that are functional for torque-based control of a bionic leg (18). However, in our study, the duration of the training sessions of AMI participants was shorter (Tables S8 and S9) compared to the 6 training trials mentioned in the literature (18, 19). In our training protocol, participants completed 20–40 min with the Empower OFF and up to 80 min with the Empower ON (Tables S8 and S9). In comparison, Ref. (18) reported ∼3 h of training per participant, while Ref. (19) reported up to 6 hours. Nevertheless, AMI participants were able to complete the trials as intended. The higher learning rate of this patient group is likely attributed to proprioceptive enhancements resulting from the combined effects of AMI and TSR surgeries, and it might also have been influenced by the implementation of a musculoskeletal model-based controller (30). However, the training duration was likely insufficient for subject 3-BAP, who struggled to maintain performance once visual feedback was removed, as he might have experienced cognitive fatigue.

Our proposed approach differs from previously proposed model-based control methods in several aspects. In Ref. (15), a linear mapping between muscle activations and torques was applied at the prosthetic joint. This mapping was calibrated heuristically at the beginning of the experiments (15). Resulting decoded torques were applied to a virtual joint, which was then simulated in terms of stiffness, damping, and inertia, finally resulting in position and stiffness setpoints to the bionic leg. Such a method would not fully account for the nonlinearities between muscle electrical signals and generated torques, and chosen stiffness and damping values represent a generic participant or were heuristically determined. Similarly, previous work proposed EMG-based ankle bionic leg control based on the modulation of powered plantarflexion (17, 18) and, more recently plantar-dorsiflexion (16, 47) with a proportional mapping between measured EMGs and torques. In this, the pneumatic nature of their bionic leg allowed them to set the baseline ankle stiffness. Furthermore, Shu et al. (21) drove a neuromuscular model of the amputated leg with surface EMGs. Torque profiles generated as outputs from a reflex-based musculoskeletal model were used as references for calibrating musculoskeletal model parameters in a second step, which in turn was driven by surface EMGs of the residual limb. However, their approach used such a model only during powered plantarflexion at the end of the stance phase, employing nonvolitional impedance control otherwise, thereby restricting bionic leg volitional control to a subphase of the gait cycle. Moreover, in none of these studies, the underlying musculoskeletal model was established to match the anatomy and force-generating properties of the amputee’s intact leg.

More recently, (19, 20) utilized EMG signals from an AMI muscle pair (TA and GM) to estimate a target joint position and impedance, while modulating maximum joint torques based on joint angle and velocity (19), akin to the behavior observed in biological joints. The primary distinction from our approach lies in the incorporation of a model for the missing limb and the personalization of the joint to the individual.

Musculoskeletal models have been explored for bionic leg control in a few studies (6, 21), where model personalization was based on reference data from healthy populations (6) or achieved through simulation environments (21). Our study introduced a musculoskeletal model that directly replicates the contralateral intact limb of a person with amputation, offering an individualized and physiologically relevant approach. Previous literature indicated that the AMI surgery could enable more effective and biomimetic sensorimotor control postamputation (48), which could promote embodiment and usability of the neural control (4, 7). The fast learning in the training sessions compared to previous work (18), and drastic improvement during bionic leg control (see Fig. 4) shown in this study confirmed these findings. A primary limitation of our study was the involvement of a small number of participants. Therefore, the statistical analysis considered conditions within the same subject. Further investigation is required to involve a larger population, including individuals with non-AMI amputations, as well as those undergoing AMI procedures and BAP. Furthermore, future research is necessary to understand if our approach could be extended to different walking conditions, including also movements such as ramps, stairs, or out-of-the-lab settings. The need for including a bio-feedback based training session (Fig. 4, Results-Gait training via muscle visual feedback section, and Video S4), as documented in prior literature (49, 50), requires further exploration to determine its necessity specifically for AMI amputees compared to other patient groups (18).

The imposed electrical constraints on the bionic leg affected participant performance, as the delivered torques were limited to 50–70 Nm, below the physiological expectations for the participant’s weight. When no measures were in place to prevent the hardware end-stops from being reached during active control, this could cause physical damage to the bionic leg. Such a limitation was overcome by a software-based end-stop, allowing the limit to be increased to 70 Nm. Moreover, the EMPOWER parallel passive spring design limited the range of effective active torque control of the bionic leg. Future studies are needed to evaluate the benefit of this type of control while providing higher torques. In terms of utility and embodiment, we recognize the importance of evaluating our technology by assessing user learning rates, cognitive load, trust in the bionic leg, satisfaction, and sense of embodiment. It is, therefore, desirable to include objective and subjective metrics evaluating those aspects in future studies.

We did not aim to investigate the long-term performance of the proposed system. Calibration was maintained throughout our trials by reperforming MVC measurements at the beginning of each day. Nonetheless, factors such as electrode degradation or variability in electrode placement could potentially affect signal quality and, consequently, system calibration. Future studies should systematically evaluate the stability and robustness of system performance over extended periods of use. Moreover, the NMBC is unlikely to overcome all the inherent challenges traditional amputees face in activating residual muscles (co-contraction, fatigue, and missing proprioception).

## Supplementary Material

pgaf413_Supplementary_Data

## Data Availability

Data required to replicate the results obtained in this article are available via the DOI 10.5061/dryad.qbzkh18rx.

## Previous Presentation

These results were previously presented at [RehabWeek, 2023 September 24–28].

## Preprints

A preprint of this article is published at 10.5281/zenodo.15096763.
