# Supplementary material for: Continuous volitional control of a bionic leg supports diverse walking patterns in both agonist–antagonist muscle interface and bone-anchored prosthesis users
Source: PNAS Nexus. 2026 Jan 5;5(1):pgaf413. doi: 10.1093/pnasnexus/pgaf413 (PMC12851846; doi:10.1093/pnasnexus/pgaf413)
Supplement: pgaf413_Supplementary_Data [file pgaf413_supplementary_data.zip › PNASNEXUS-PNASNEXUS-2025-00357R-s10.pdf]

Supplementary Materials

Supplementary Materials and Methods

The protocol involved a 3-day session at the University of Twente (Enschede, The Netherlands). Fig.1 gives an overview of the real-time walking protocol at ground elevation. Day 1 was dedicated to the bionic leg alignment, the selection of adequate positions of the electrodes, and the first training on how to use the myoelectric controller (Video S4, Fig. 4). We placed 8 monopolar electrodes on both muscle bellies of the gastrocnemius medialis (GM) and tibialis anterior (TA). In the case of Subject 3-BAP, only 4 electrodes were placed since the residuum was smaller (Fig. 1). Further, we selected one pair of electrodes that yielded a bipolar signal, and we chose one signal channel per muscle by analyzing the activities of each group during walking. Days 1 and 2 were dedicated to the training of the participant while walking at their preferred speed. A graphical user interface (GUI) based on previous research [29] provided the participant with visual feedback of one of his muscle activations at a time over the gait cycle, alternating sessions with GM feedback with sessions with TA feedback. The participant was instructed to follow a reference profile for the GM and TA muscles individually. Reference profiles were derived from our previous work on human locomotion and muscle modularity [25], which were adjusted in amplitude and peak time to best match his preferred pattern. On day 1, each user performed the trials with the passive prosthesis. On day 2, Subjects 2-AMI and 3-BAP could already experience the active bionic leg. Data collected on the training of day 1 were employed in creating the participant-specific neuromechanical model (*i.e.*, muscle activations, leg joint angles, and reference ankle joint torques from the intact limb). See Section Materials and Methods - Model calibration for more details on the model calibration procedure. The number of training trials followed by each participant can be seen in Tables S8 and S9. Day 3 focused on the actual trial, where each subject was challenged to complete varied trials with the bionic leg driven by a personalized neuromechanical model-based control (NMBC):

1. **Subject 1-AMI** performed calf-raises while following the cadence of a metronome at 3 different frequencies (30, 45, and 60 beats per minute (bpm) - Video S2, S3, Fig. S6) and, ground-level treadmill walking at 3 different speeds (1.6 km/h, 2km/h, and 2.4 km/h - Video S1, Figs 2,3)
2. **Subject 2-AMI** performed ground-level treadmill walking at 3 different speeds(1.98 km/h, 2.34km/h, and 2.69 km/h - Video S5, Fig. 3), and treadmill walking at preferred speed at three different ground inclinations (ground, 3% and 5% - Video S6, Fig. S4).
3. **Subject 3-BAP** performed ground-level treadmill walking at 3 different speeds (2.52 km/h, 3.24 km/h, and 3.96 km/h - Video S7, Fig. 3), and treadmill walking at preferred speed at three different ground inclinations (ground, 3%, and 5% - Video S8, Fig. S4).

Experimental protocol

The complete experimental session spanned 3 days. The detailed protocol performed by Subject 1-AMI is provided below. The first 2 days involved a training session based on visual feedback of electromyography (EMG) signal-based activation, during which the prosthesis was powered OFF. On the third day, the participant controlled the ankle prosthesis using our proposed EMG control framework during different movements

Day 1 - Prosthesis alignment and Channel selection

- Alignment of the prosthesis:
  A certified prosthetist from the Roessingh Rehabilitation Center performed a static and dynamic prosthesis alignment.
- EMG activation channel selection
  - Placement of 8 bipolar electrodes (4 in case of Subject 3) to cover the entire belly of 2 muscles of the residual limb, namely the GM and TA.
  - The participant was instructed to activate the TA and GM of the residual limb alternately while sitting and not wearing his socket.
  - The participant was instructed to activate the TA and GM of the residual limb alternately while sitting and wearing his socket.
  - Walking trial with visual feedback to choose the channel for each muscle:
    The trial was meant to select one EMG signal channel from each muscle of interest among $c = \{GM, TA\}$ that most approximate the activation patterns of intact limb individuals. The use of visual feedback of one selected EMG signal channel at a time allows us to verify the controllability over the muscle contraction given a reference curve to follow. Moreover, this allowed us to collect preliminary data useful for the calibration of the model, and it functioned as a first training session.

Day 2 - Adaptation with unpowered prosthesis
The session had the purpose of evaluating if the participant could re-learn muscle excitation patterns following a predefined pattern.

- EMG activation channel selection
  - Placement of EMG electrodes
  - Walking trial at preferred speed
    The trial was meant to select one EMG channel from each muscle of interest among $c = \{GM, TA\}$ that most approximate the activation patterns of intact limb individuals.
- Maximum voluntary contraction (MVC) measurement
  Maximum effort isometric plantarflexion and dorsiflexion while sitting and standing for 10 times in each direction. Maximum values were used for the normalization of EMG activation signals.
- Walking at self-selected speed with visual feedback of GM
  The participant walked on the treadmill at his preferred speed. The goal of the trial was to adapt to our interface and learn how to modulate his muscle contraction following the reference. This trial was repeated twice with a duration of 10 minutes each. The trial was interrupted earlier if the participant expressed fatigue.
- Walking at self-selected speed with visual feedback from TA
  Same as for the GM

Day 3 - Adaptation and Walking trial

- Instrumentation
  Placement of EMG electrodes and 37 motion capture markers
- Bilateral calf raise under metronome with the prosthesis ON
  For this test, the participant performed one full cycle of the calf raise movement per beat, therefore at the beep, the participant would apply torque at the ankle and raise from the ground, hold the plantar-flexed position for a fraction of a second, and then return to the natural position with the heel on the ground. The metronome was set for cadences of 30, 45, and 60 beats per minute (bpm). The participant was

not able to follow the 60bpm cadence, therefore results are not included.

- Treadmill walking at 3 different speeds with the prosthesis ON
  The participant walked at his preferred speed ($v_0 = 2km/h$) for 45 seconds, at speed $v_1 = 1.2 \times v_0 = 2.4km/h$ for 45 seconds and at speed $v_1 = 0.8 \times v_0 = 1.6km/h$ for 45 seconds.
- Treadmill walking at 2 different speeds with the prosthesis ON
  The participant walked at his preferred speed ($v_0 = 2km/h$) for 45 seconds and at speed $v_1 = 1.2v_0 = 2.4km/h$ for 45 seconds.

NMBC Calibration

Muscle-tendon unit (MTU) strength coefficients (Fig. S1-stage D) varied within the range of 0.5 to 1.5 to scale the maximal isometric force nominal values for all dorsiflexing and plantarflexing muscle MTUs [17]. This scaling preserved physiological force ratios within the 2 muscle groups while matching the individual's force-generating capacity.

Moreover, the tendon slack length and optimal fiber length (Fig. S1-stage D) were respectively adjusted within ±8% and ±3% of their initial values (from the first optimization step).

Data collection

A motion capture system provided marker data in 3D (Oqus, Qualisys, Sweden), and an instrumented treadmill provided 3-dimensional foot-ground reaction forces (GRFs), torques, and center of pressure (M-Gait, MotekForce Link, The Netherlands). EMG signals were collected by 8 bipolar channels placed along 2 muscles (GM, TA) on the residuum (4 for Subject 3-BAP). To guarantee user comfort with the socket in place, we used disposable adhesive gel electrodes (disposable adhesive 4-disk electrodes from Technomed Europe) and Myoplus amplifiers (Myoplus, Ottobock, Germany). Each muscle was instrumented with 4 (2 for Subject 3-BAP) bipolar channels, but only the muscle activation channel that represented a unilateral contraction with the least noise level was used for control.

Data Processing

Online data refers to the data available during the experiments, which informs the control of the bionic leg. Offline data, on the other hand, were logged and analyzed after the experiment.

Online Data Processing Pipeline

EMG data passed through a Butterworth band-pass filter of cutoff frequencies 30 and 300 Hz, then their absolute value was taken, and finally, the envelope was determined by filtering it with a Butterworth 2nd order low-pass filter with a cutoff frequency of 3Hz. Prosthesis angle data were filtered by a Butterworth 2nd order low-pass filter with a cutoff frequency of 3Hz. The delivered torques were limited to 50 and 70 Nm. This limit was set as a safety measure implemented due to software constraints. In particular, no measures were in place to prevent the hardware end-stops from being reached during active control, which in turn could cause physical damage to the bionic leg. A software constraint was implemented for Subject 3-BAP's experiment and the torque limit was increased to 70Nm. This limitation affected the amount of support generated by the bionic leg during push-off.

Offline Data Processing Pipeline

Marker data were preprocessed within the manufacturer's proprietary software (QTM, Qualisys, Sweden), where marker gaps were filled, and subsequently, data were exported and loaded into MATLAB (The MathWorks Inc., United States). Marker and GRF data were filtered by a fourth-order zero delay low pass Butterworth filter with a cutoff frequency of 20Hz. Data logged in TwinCAT (EMGs, GRFs) were loaded directly into MATLAB. Data from different sources were synchronized by an external trigger signal generated by the TwinCAT master. All online data were also saved for use during offline analysis, which was performed in MATLAB.

Assessment of the outcomes

- The analysis of speed adaptation during over-ground walking and calf-raises relied on the time between peaks of the signals of interest. EMG peaks were times between peaks of muscle activations during a given movement. Peaks were obtained by aggressively filtering the EMG signal with zero delay (offline) and detecting local maxima. Only peaks above a threshold value were considered. Torque peaks were detected in the same manner. The time distances are calculated as the time difference between two peaks, and it is reported as the mean and standard deviation. Statistical analysis was conducted using the Wilcoxon rank sum test. The tests were performed for each time variable across different speed categories: slow to medium, medium to fast, and slow to fast. The Bonferroni correction was applied to adjust p-values considering 3 pairwise tests for each subject[7]. Moreover, the time of both GM and TA activation peaks and NMBC torque peaks along each gait cycle were compared to reference patterns from the literature on intact-limb biomechanics [48].
- The gait training sessions evaluated if the participant could re-learn the timing of peak muscle activations by following reference targets provided to the user via visual feedback (Fig. 4). The goal was to give each participant time to adapt his residual muscle activation patterns to be functional for the control of the bionic leg [29]. To analyze the results we defined plantarflexion activation peaks to be within the target range if they fell within the mid-to-late stance phase *i.e.*, between 30% and 60% of the gait cycle. Similarly, dorsiflexion activation peaks were considered within the target range if they fell within the early-to-mid swing, *i.e.* between 60% and 90% of the gait cycle. The percentage of muscle activation peaks in the range is determined for each experimental trial. Correlation values R between GM and TA activation patterns and healthy activation patterns from literature [49] were calculated on Day 1 and Day 3 of the experiment.
- We evaluated the accuracy of the participant in modulating the amplitude of muscle activation peaks and active bionic leg peak torque across all inclinations throughout the entire test duration. EMG and torque peaks were detected in the same manner as previously explained.

Figures and Tables

Table S1. Time between peaks of filtered and normalized EMGs and bionic leg joint torques in the plantarflexion and dorsiflexion directions, for the walking speeds of 1.6, 2 and 2.4 km/h in Subject 1-AMI. Results are reported in seconds (s) as mean±standard deviation.

	1.6km/h		2km/h		2.4km/h	
Signal	PF (s)	DF (s)	PF (s)	DF (s)	PF (s)	DF (s)
EMGs	1.8±0.2	1.5±0.5	1.7±0.1	1.6±0.3	1.5±0.1	1.5±0.1
Torques	1.8±0.2	–	1.5±0.3	–	1.4±0.2	–

Table S2. Time between peaks of filtered and normalized EMGs and bionic leg joint torques in the plantarflexion and dorsiflexion directions, for the walking speeds of 1.98, 2.34 and 2.69 km/h in Subject 2-AMI. Results are reported in seconds (s) as mean±standard deviation.

	1.98km/h		2.34km/h		2.69km/h	
Signal	PF (s)	DF (s)	PF (s)	DF (s)	PF (s)	DF (s)
EMGs	1.8±0.2	1.9±1	1.7±0.1	1.8±0.7	1.5±0.1	1.7±0.7
Torques	1.7±1.2	–	1.8±0.7	–	1.5±0.8	–

Table S3. Time between peaks of filtered and normalized EMGs and bionic leg joint torques in the plantarflexion and dorsiflexion directions, for the walking speeds of 1.6, 2 and 2.4 km/h in Subject 3-BAP. Results are reported in seconds (s) as mean±standard deviation.

	2.52km/h		3.24km/h		3.96km/h	
Signal	PF (s)	DF (s)	PF (s)	DF (s)	PF (s)	DF (s)
EMGs	1.6±0.8	1.7±1.6	1.5±1.2	1.5±0.8	1.4±0.8	1.5±0.8
Torques	1.5±0.8	–	1.3±0.6	–	1.2±0.7	–

Table S4. Correlation coefficient between transtibial amputee participants' and intact-limbed individuals' filtered and normalized EMGs for gastrocnemius medialis (GM) and tibialis anterior (TA)

R	GM-Day 1	GM-Day 3	TA-Day 1	TA-Day 3
Subject 1-AMI	0.53	0.94	0.66	0.03
Subject 2-AMI	0.32	0.86	0.01	-0.18
Subject 3-OI	0.36	0.07	-0.04	0.47

Table S5. Mean $\pm SD filtered and normalized EMG amplitude of gastrocnemius medialis (GM), tibialis anterior (TA) as well as prosthesis joint torques in the$ [illegible]
AMI

	ground	3%	5%
GM	0.32±0.17	0.38±0.12	0.38±0.12
TA	0.32±0.17	0.38±0.12	0.63±0.19
active prosthesis torque	-29.5±17.4	-37.7±16	-40.7±17.1

Table S6. Mean $\pm SD filtered and normalized EMG amplitude of gastrocnemius medialis (GM), tibialis anterior (TA) as well as prosthesis joint torques in the$ [illegible]
OI

	ground	3%	5%
GM	0.53±0.29	0.23±0.23	0.18±0.28
TA	0.18±0.12	0.25±0.22	0.09±0.13
active prosthesis torque	-35.2±23.7	-14.2±8.2	-29.1±26.3

Table S7. Time (s) between peaks of filtered and normalized EMGs, prosthetic joint angles, and active prosthesis torques in the plantarflexion direction, for the metronome cadence of 30 and 45 beats per minute (bpm) in the calf raise exercise. Results are reported as mean±standard deviation.

Signal	30 bpm	45 bpm
EMGs	2.0±0.1	1.4±0.1
Torques	2.0±0.5	1.4±0.1
Angle	2.7±3.5	1.6±1.2

Table S8. Number of training sessions, mean number of trials per session, maximum trial duration (minutes), and total duration (min) of the training trials with EMPOWER OFF.

	Sessions (Count)	Trials/Session (Mean)	Minutes/Trial (Max)	Total (minutes)
Subject 1-AMI	2	1.5	10	30
Subject 2-AMI	1	4	10	40
Subject 3-OI	1	2	10	20
Average	1	2.5	10	30

Table S9. Number of training sessions, mean number of trials per session, maximum trial duration (min), and total training duration (min) with EMPOWER ON.

	Sessions (Count)	Trials/Session (Mean)	Minutes/Trial (Max)	Total (minutes)
Subject 1-AMI	0	0	0	0
Subject 2-AMI	1	2	10	20
Subject 3-OI	1	8	10	80
Average	1	3	7	30

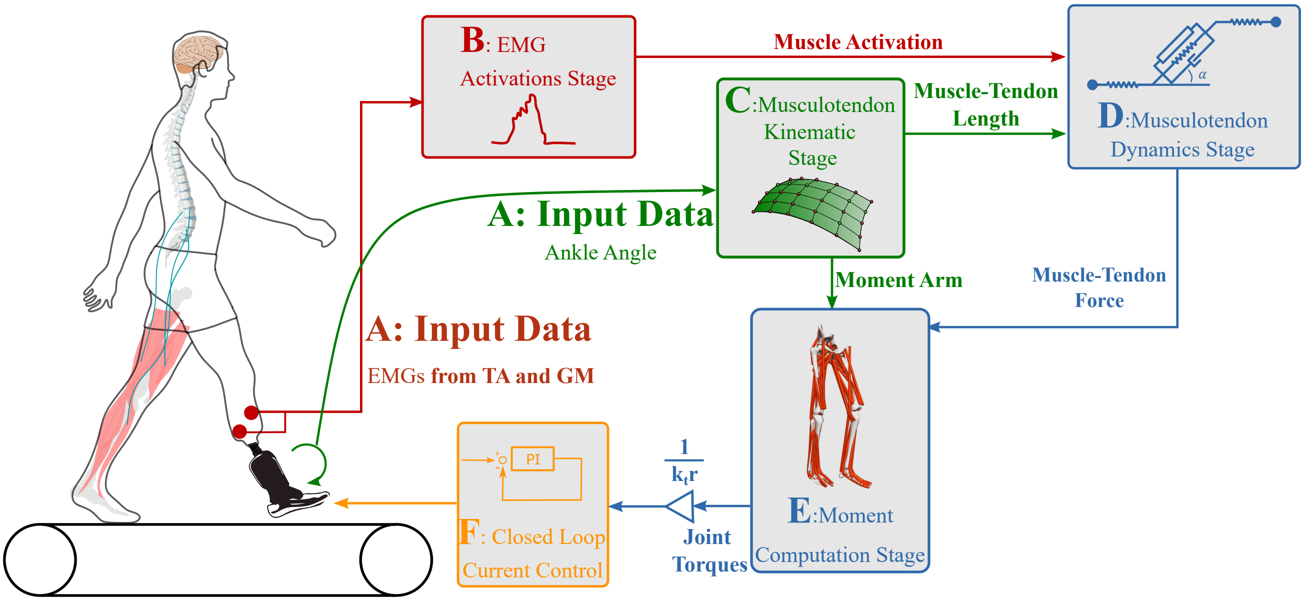

Figure S1. Schematics of the EMG-driven-Neuromechanical Model-Based controller (NMBC), calibrated from participant-specific data of the intact leg (B-E) [36]. EMG-derived activations and joint angle data (A) are inputs to the model. EMG are filtered and amplitude-normalized (B) to obtain activation envelopes (details in the methods section) and further processed via a participant-specific, non-linear, excitation-to-activation shape factor. The Musculotendon Kinematic Stage (C) synthesizes subject-specific paths of muscle-tendon units (MTUs) by utilizing a set of MTU-specific cubic B-splines. Each B-spline calculates the MTU length and moment arms based on the input joint angles. The Musculotendon Dynamics Stage (D) determines the dynamic equilibrium between muscle fibers and series-elastic tendons to produce the net MTU force. It utilizes a Hill-type muscle model, with a series elastic tendon, which was assumed to be massless. The model has inherent elastic properties (dictated by the active and passive force-length and force-strain relationships in modelled muscles and tendons) as well as damping properties (i.e., dictated by the force-velocity relationship with parallel damping) as previously described in our previous work [34]. During a dynamic movement, the MTU forces are derived from estimated muscle fiber length, muscle fiber contraction velocity, and the state of activation of the muscle fiber. The Moment Computation Stage (E) transfers MTU forces to the skeletal joint level using MTU moment arms. The motorized prosthesis operates in closed-loop current control (F). Torque commands are converted into current commands via the motor constant (k_t) and transmission ratio (r).

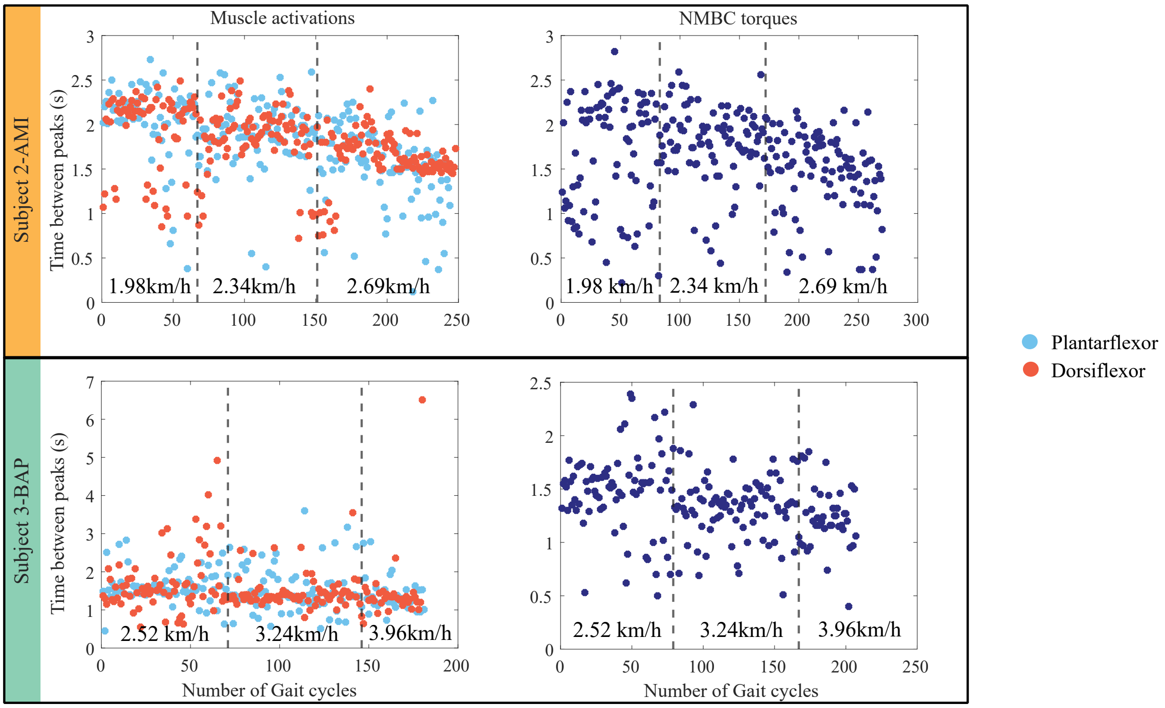

Figure S2. Time in seconds between peaks of muscle activations for gastrocnemius medialis (GM, blue), tibialis anterior (TA, red) and active prosthesis torques, during the walking trial on a treadmill on the second day of experimental sessions.

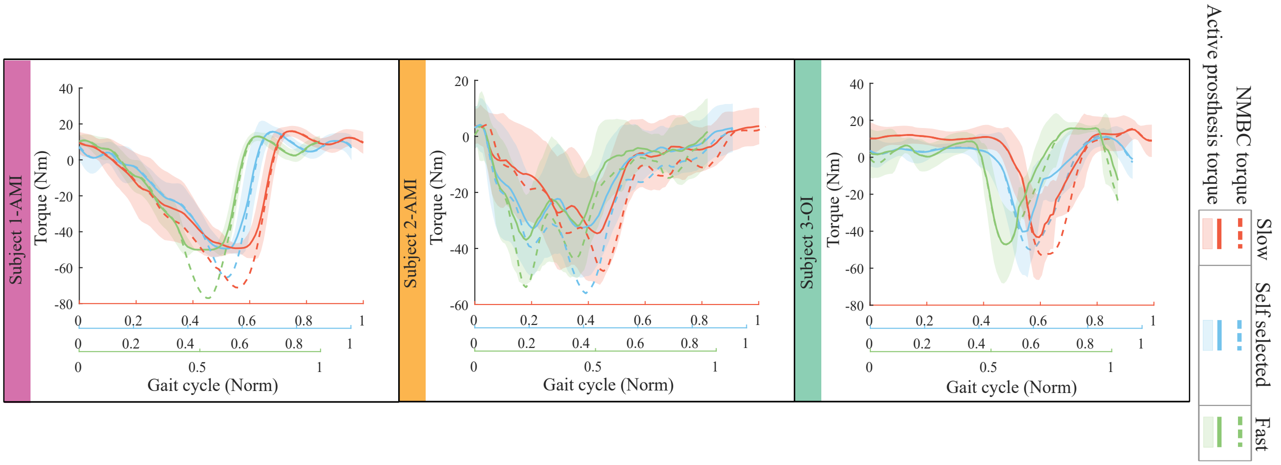

Figure S3. Joint torques at different speeds. Solid lines indicate averages and, shaded areas standard deviations of the bionic leg torques. Dashed lines are EMG-driven neuromechanical model-based control (NMBC)-decoded torques (more details in section Materials and Methods). Each participant performed treadmill walking at three different speeds: slow (red), medium (blue), and fast (green). Normalized gait cycles (x-axes) are scaled by their relative duration.

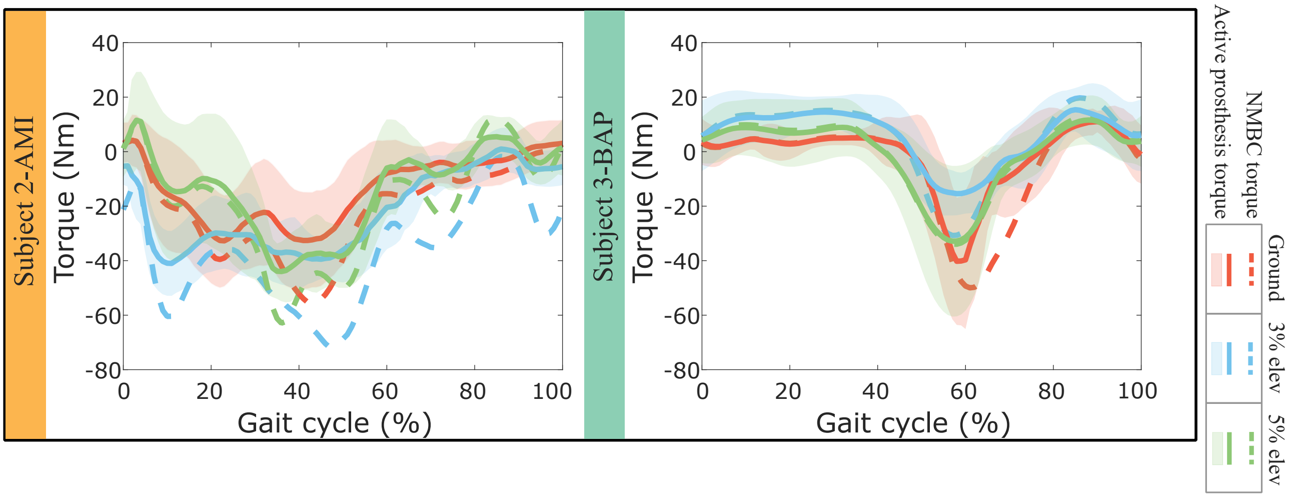

Figure S4. Joint torques at different ground elevations. Solid lines indicate averages, and shaded areas illustrate standard deviations of the active prosthesis torques. Dashed lines are EMG-driven neuromechanical model-based control (NMBC)-decoded torques (more details in section Materials and Methods). Each participant performed treadmill walking at three different elevations: ground (red), 3% elevation (blue), and 5% elevation (green).

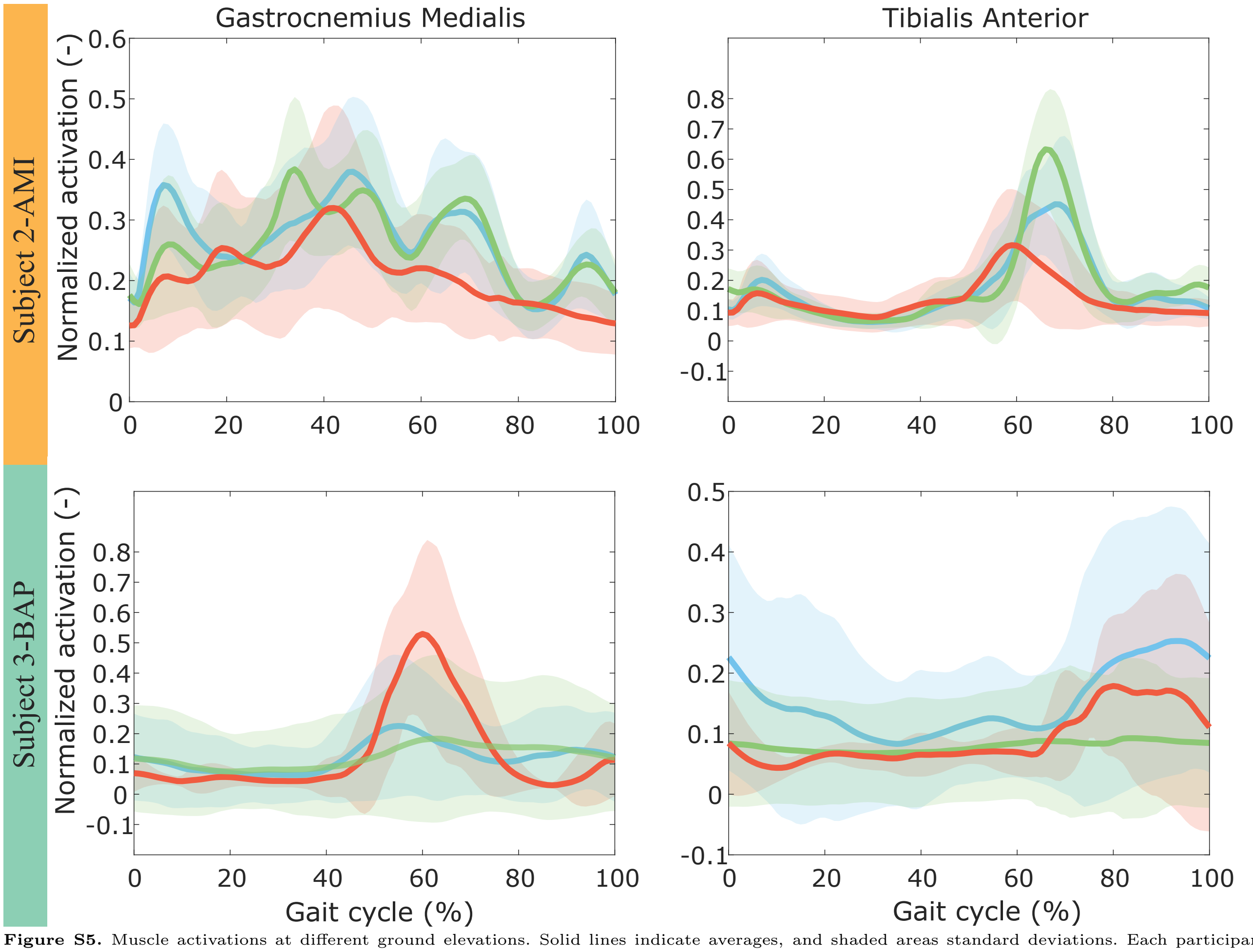

Figure S5. Muscle activations at different ground elevations. Solid lines indicate averages, and shaded areas standard deviations. Each participant performed treadmill walking at three different elevations: ground (red), 3% elevation (blue), and 5% elevation (green).

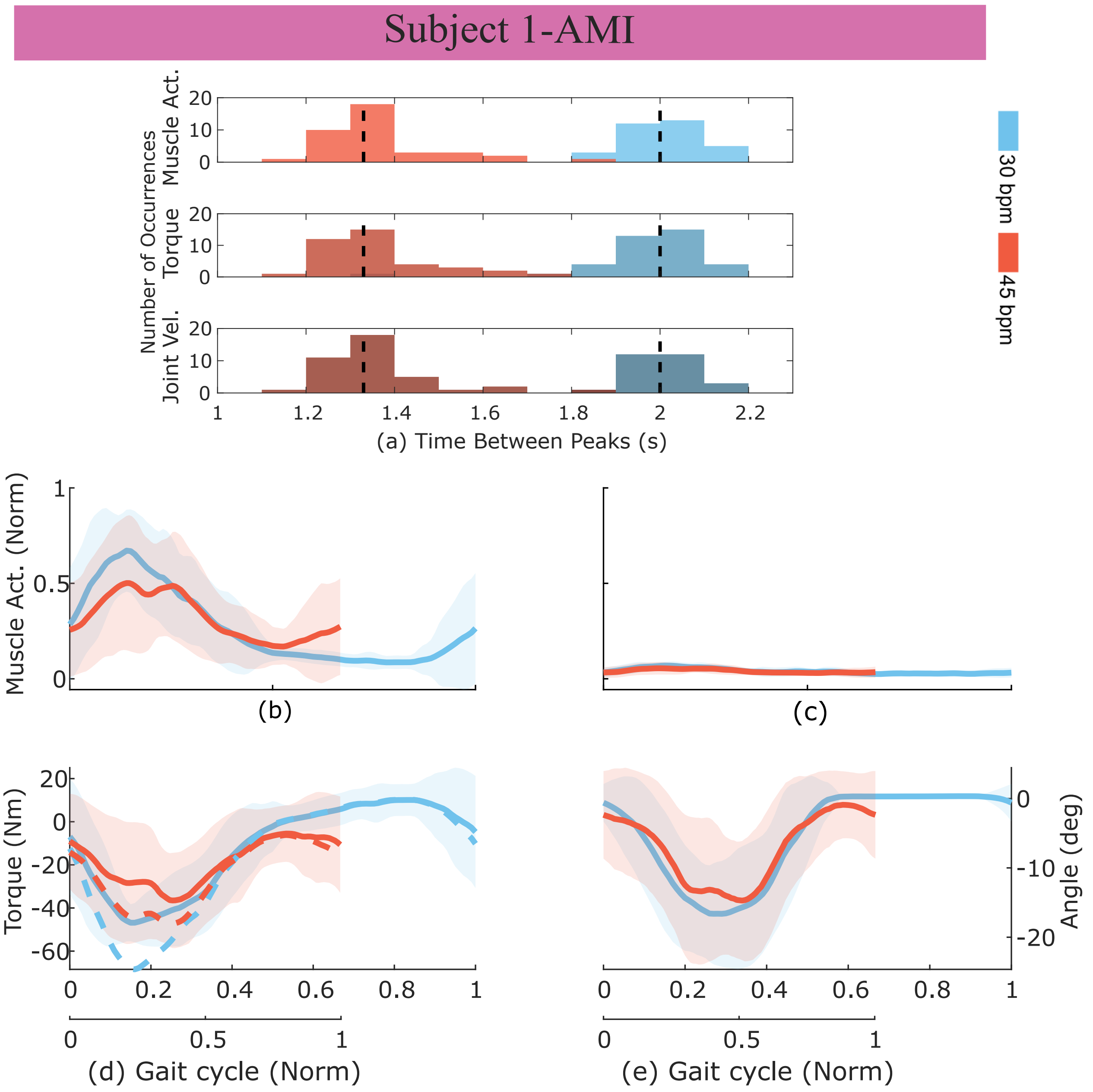

Figure S6. Histograms of peak muscle activations (row 1), torques (row 2), and peak angular velocities (row 3) of the prosthetic ankle joint of plantarflexors (or plantarflexion) during metronome dictated calf raise task for cadences of 30 and 45 beats per minute (bps) on the third day of experiments (**a**) of Subject 1-AMI. Muscle activations of gastrocnemius medialis (GM) (**b**) and tibialis anterior (TA) (**c**), as well as active prosthesis ankle torques (**d**) and angles (**e**), are also depicted. Dashed lines are neuromechanical model-based control (NMBC) torques unconstrained by the limits of the prosthesis. The participant performed a plantarflexion at each beep and returned to the natural position with heels on the ground.
