# Supplementary material for: Continuous volitional control of a bionic leg supports diverse walking patterns in both agonist–antagonist muscle interface and bone-anchored prosthesis users
Source: PNAS Nexus. 2026 Jan 5;5(1):pgaf413. doi: 10.1093/pnasnexus/pgaf413 (PMC12851846; doi:10.1093/pnasnexus/pgaf413)
Supplement: pgaf413_Supplementary_Data [file pgaf413_supplementary_data.zip › PNASNEXUS-PNASNEXUS-2025-00357R-s01.pdf]

Brief Description of the Videos

- **Video S1.** Level-ground walking at three different speeds, performed by Subject 1-AMI.
- **Video S2.** Calf raises at 45 beats per minute (bpm), performed by Subject 1-AMI.
- **Video S3.** Calf raises at 30 bpm, performed by Subject 1-AMI.
- **Video S4.** Walking training with visual feedback, performed by Subject 1-AMI.
- **Video S5.** Walking at three different speeds, performed by Subject 2-AMI.
- **Video S6.** Walking at two inclinations, performed by Subject 2-AMI.
- **Video S7.** Walking at three different speeds, performed by Subject 3-BAP.
- **Video S8.** Walking at two inclinations, performed by Subject 3-BAP.
